# Technology Supporting Cognitively Impaired Older Adults: A Scoping Review for the ENHANCE Center

**DOI:** 10.1093/geroni/igab046.2635

**Published:** 2021-12-17

**Authors:** Edie Sanders, Robin Stuart, Alexander Exum, Walter Boot

**Affiliations:** Florida State University, Tallahassee, Florida, United States

## Abstract

Cognitive impairments (CIs) result in difficulties with a wide range of daily activities. Older adults are especially at risk for CIs, and as the older adult population increases, so does the importance of understanding and supporting the needs of those with CIs. The Enhancing Neurocognitive Health, Abilities, Networks, and Community Engagement (ENHANCE) Center was established with a focus on developing technology-based support for socialization, transportation, and prospective memory needs of older adults with CIs due to mild cognitive impairment (MCI), traumatic brain injury (TBI), and stroke. The extent to which relevant literature in these domains existed was unknown. We conducted a scoping review to identify existing research meeting the following criteria: participants aged 50+ years classified as having a CI due to MCI, TBI, or stroke; and a focus on technology-based support for socialization, transportation, and/or prospective memory needs. Using PRISMA guidelines, we searched three electronic databases, and reviewers screened citations for inclusion and completed data charting. Following screening, only 11 studies met our inclusion criteria. Qualitative and quantitative data are reported for each study. In addition to few studies available, it was common for studies to include 20 or fewer participants. Most assessed technology interactions at one time and few studies examined longitudinal use and benefits. While each paper examined one aspect of user-centered design, no technologies were reported that underwent all stages of the user-centered design process, from needs assessment to iterative design and usability testing, to efficacy trial. Such gaps highlight the important role ENHANCE can play.

